# The interactions of model cationic drug with newly synthesized starch derivatives

**DOI:** 10.5599/admet.1950

**Published:** 2023-09-20

**Authors:** Justyna Kobryń, Tomasz Zięba, Magdalena Rzepczyńska, Witold Musiał

**Affiliations:** 1 Department of Physical Chemistry and Biophysics, Wrocław Medical University, Borowska 211A, 50-556 Wrocław, Poland; 2 Department of Food Storage and Technology, Faculty of Biotechnology and Food Science, Wroclaw University of Environmental and Life Sciences, Chełmońskiego 37, 51-630 Wrocław, Poland

**Keywords:** potato starch, adsorption, interaction, methylene blue

## Abstract

**Background and purpose:**

The aim of the work was to compare the interactions of three newly synthesized non-toxic starch derivatives, with varied anionic and non-ionic functional groups with methylene blue (MB) as a model cationic drug, and selection of starch derivative with highest affinity to the MB.

**Experimental approach:**

The native potato starch (SN), modified *via* acetylation (SM1), esterification and crosslinking (SM2) and crosslinking (SM3), was evaluated in MB adsorption studies and assessed by FTIR, PXRD, and DSC.

**Key results:**

The adsorption of MB on SM2 and SM3 matched the BET isotherm model, which confirmed physisorption on the low-porous surface. In the case of SM1, adsorption took place *via* electrostatic attraction between the heterogeneous adsorbent surface and the adsorbate, as demonstrated by the Freundlich plot. The FTIR confirmed vibrations assigned to N=C stretching bonds at 1600 cm^-1^ in the case of MB adsorbed on the SN and SM2. The most intense PXRD peaks belonged to SN and the least to SM2. In the DSC study, the thermal stability *via* Δ*T* was assessed, with SM2 of lowest Δ*T* value (179.8 °C).

**Conclusion:**

SM2 presented the best adsorption capacity, followed by SM3 and the weakest SM1. The interactions were confirmed in the adsorption studies and may reflect applications of the modified starches as drug carriers. In the FTIR study, a probable interaction between the OH^-^ groups of SM2 and N^+^ of MB was revealed. The most amorphous structure was shown for SM2, which was correlated with the lowest thermal stability provided by the DSC study.

## Introduction

**Figure 1.**
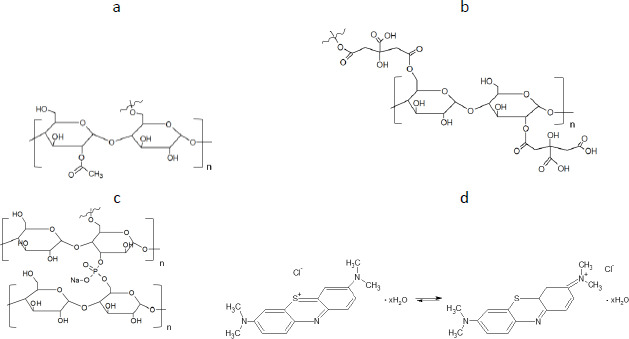
Structural models of the adsorbates as: a - starch acetate (SM1), b - starch citrate (SM2), c - starch diphosphate (SM3) and the d - adsorbent methylene blue tautomers (MB).

There are numerous newly developed carriers for therapeutic substances [1-3]. They are often completely new synthetic polymers [4,5]. Among polymers of plant origin, there are natural examples of potential carriers [6-10]. Wheat, maize and potato starch are available in large quantities from natural sources [11-13]. So far, starch has been modified by Utomo, Odeniyi, Singh *et al.* [14-16], among others. Previous publications have presented new methods for obtaining a number of starch derivatives [17-19] and proposed the potential use of some of them, including acetylated starch [20,21] and starch citrate [22]. The figure below shows the structural models of the modified potato starches created by the team from the University of Environmental and Life Science (Wrocław, Poland) (Figures 1a,b,c).

Some drugs have a 1,4-thiazine ring, which is included in the structure of methylene blue (MB). The study that used the similarity between MB and other molecules was performed on acridine and phenothiazine derivatives for anti-prion therapy [23]. Selected drugs include the 1,4-thiazine ring: phenothiazine, which has antiparasitic, antiseptic and antioxidant effects [24], promethazine, which has antihistamine and anti-allergic effects [25], chlorpromazine and [26] thioridazine - as psychotropic drugs. The ionic interaction between the promethazine and Eudragit® was ascribed to the electrostatic interaction between the drug and ammonium groups in the polymer backbone [27]. The interactions of chlorpromazine with cyclodextrin were realized *via* ionic bonds between the ionized amine group of chlorpromazine and anionic group of cyclodextrin sulfate [28]. Another study, which exploited cyclic voltammetry, interpreted the interactions of chlorpromazine and thioridazine with bovine serum albumin nanoparticles as a result of hydrophobic interaction [29]. Therefore, research has been undertaken on MB as a model drug representing the structural features of some active pharmaceutical ingredients (API). These studies may determine whether similar drugs could adsorb on modified starch. To date, studies have analyzed the adsorption of MB and other dyes on activated carbon, minerals of natural origin such as perlite, clay and its derivatives, bentonite [30,31], and agricultural solid wastes [32]. In summary, the important group that led to the intermolecular activity may be the ammonia group, which specifically interacts with anionic functionals. Because of the wide potential application of modified potato starch [33-36], we decided to compare the adsorption capacity of selected starch derivatives, characterized by a distinct negative charge of carboxyl and phosphate groups, or a characteristic ester group, towards methylene blue as a cationic substance.

The aim of the work was the evaluation of the prospective pharmaceutical applicability of three newly synthesized non-toxic starch derivatives, assessed *via* analysis of interactions of the starches, modified by varied acidic and non-ionic functional groups, with methylene blue, as a model cationic drug. The main question was the selection of the starch derivative with the highest affinity to the MB molecule. The possible interactions were evaluated in the terms of spectroscopic structural studies, as well as thermal assessments. The interactions, which may lead to drug release prolongation, were practically confirmed in the adsorption studies, which may reflect use of the above-mentioned modified starches as drug carriers for topical application, with retardant and prolonging effect against drug release process.

## Materials

The following materials were employed in the study: methylene blue (MB, Reko, Dzierżoniów, Poland) - a model active pharmaceutical ingredient (API), native potato starch (SN) (PPZ, Niechlów, Poland), modified potato starches (starch acetate - SM1, starch citrate - SM2 and starch diphosphate - SM3) prepared according to below-described methods.

## Methods

### Preparation of the modified starches

The thermal and chemical modifications of native potato starch were conducted and resulted in acetylation (SM1), esterification and crosslinking (SM2), and crosslinking (SM3).

### Preparation of acetylated starch (SM1)

The fractions of starch particles with an average volume moment diameter *D* [3,4] equal to 39.1 and 61.7 μm were separated from the native potato starch (SN) by measuring the volume diameter (Malvern laser particle size analyzer). Larger starch particles were acetylated with acetic anhydride in the amount of 13 cm^3^ /100 g of starch. After drying the acetylated starch, an initial gelatinization temperature of 49.17 °C was determined by differential scanning calorimeter Flash DSC (Mettler Toledo, Poland). The acetylated starch has been stirred for 24 h at 48 °C. It was then washed three times with five-liter portions of distilled water and separated from the slurry using the Contifuge Stratos (Heraeus, Germany) flow centrifuge. The obtained starch was dried for 24 h in an air dryer at 30 °C.

### Preparation of citrate starch (SM2)

Native potato starch (SN) was esterified with citric acid. 10 g of citric acid per 100 g of starch dry matter was dissolved in 90 mL of water and thoroughly mixed. The resulting starch paste was left for 12 h at room temperature and then dried in an air dryer (Memmert, Germany) at 50 °C for 12 h. The starches were calcined for 3 h at 100 °C. The sample was rinsed three times with ethyl alcohol with a concentration of 95 mL of ethanol per 100 mL of solution, and each time, the solution was poured over the sediment. The washed precipitate was dried in an air dryer at 30 °C for 12 h. The obtained citrate starch was hardened at a temperature 2 degrees below the gelatinization temperature (determined by DSC), dried in an air dryer at 30 °C, ground in a laboratory mill and passed through a sieve with a mesh size of 400 μm [17].

### Preparation of diphosphate starch (SM3)

Native potato starch (SN) (94.6 wt.%), sodium trimetaphosphate (1.2 wt.%), sodium carbonate (2.1 wt.%) and sodium chloride (2.1 wt.%) were introduced into the reaction vessel, 500 mL of distilled water at 45 °C was added. The mixture was adjusted to pH 10.5 with a 3 % NaOH solution. It was kept at 45 °C for 30 minutes with constant stirring, then neutralized with 8 % HCl to pH 6.5-6.8. The cross-linked starch was washed several times with distilled water on a funnel under vacuum and dried for 48 h at 25 °C. After obtaining starch diphosphate, it was hardened for 24 h at 2 degrees below the gelatinization temperature, dried in an air dryer at 30 °C, milled and sieved through a sieve with a mesh size of 200 μm.

### Determination of the potential number of functional groups of modified starches

Based on the recipe for preparing individual modified starches, the mass values of substrates attached to native starch (SN), such as acetic anhydride, trimethaphosphate and citric acid, were converted to moles. The converted values per gram of SN are given in Table 1.

### Spectroscopic evaluation

#### Fourier transform infrared spectroscopy (FTIR)

Fourier-transform infrared spectroscopy (FTIR) and attenuated total reflectance (ATR) appetizer (Nicolet 380 FTIR, Thermo Scientific, Waltham, MA, USA) with OMNIC ™ software were used to determine possible interactions between MB particles and starch. The formulations of MB adsorbed on every 50 mg of starch (SN, SM1, SM2 and SM3) were dried at 40 °C and compared with a physical mixture of MB and starch in a weight ratio of 1:10 and with the standards of the pure substances. The spectra of powders were recorded at wavelengths of 400 to 4000 cm^-1^ at 32 scans per sample and a resolution of 4 cm^-1^.

#### Powder X-ray diffraction (PXRD) analysis

The analysis of starch was supplemented by X-ray diffraction measurements (PXRD). The powder PXRD data were recorded on a Bruker D2 PHASER diffractometer (Bruker AXS, Karlsruhe, Germany) with a Lynxeye detector using Cu Kα radiation (0.15418 nm). All samples were measured at 295 K with 3.0 mm slit and 1.0 mm shutter. Diffractograms were obtained between 7.5° and 40° (2) (step size of 0.02° (2) and 0.25 s per step). The X-ray generator operated at 30 kV and 10 mA. The PXRD patterns were processed using the software Diffrac.Eva V 3.2. (Bruker AXS). The percentage of crystallinity and amorphousness of the tested starch samples in relation to SN was determined.

### Thermal analysis

#### Differential scanning calorimetry (DSC)

Differential scanning calorimetry (DSC 214 Polyma, Netzsch, Selb, Germany) was performed to investigate the samples of dried adsorbed MB on 50 mg starches (SN, SM1, SM2, SM3), their physical mixtures of 1:10 w/w and pure ingredients. The grated samples of 3 to 5 mg were explored in aluminium pans with lids under a nitrogen atmosphere, with a flow rate of 50 mL min^-1^. The thermograms were recorded at a constant heating rate of 5 °C min^-1^ in the temperature range from 0 to 350 °C.

#### Evaluation of the adsorption kinetics of MB on the modified starches

The absorbance study was performed using four 50 mL conical flasks (A, B, C, D) as a series. 40 mL 6 mg L^-1^ MB was added for each flask. The SN, SM1, SM2 and SM3 starch probes of 2, 5, 10, 25, 50, 125, 250, 500, 750 and 1000 mg were placed in the flasks during the series, respectively. The flasks were put on the orbital shaker (at 100 rpm) at 22±0.5 °C. Four measurements of absorbance were proceeded based on the pharmacopoeial method by sampling the MB solution volumes of 3 mL in every 5, 10, 15, and 20 minutes, and than in equal periods up to 4 h. The solution was returned to the flasks. Analysis of taken samples was done by the spectrophotometer UV/VIS Jasco V-530 (Tokyo, Japan) at 663 nm, according to the available bibliography [37-39], and compared to the absorption spectrum of MB. A standard curve based on three series of measurements with five concentration points from 0.5 to 5.0 mg L^-1^ was prepared. The results were examined according to pseudo-zero-order kinetics and first-order kinetics. ANOVA statistical test was performed for independent groups at the significance level *α* = 0.05. Freundlich, Langmuir and BET isotherm models were taken for analysis of adsorption processes (Table 2). BET isotherm equation was adapted to form BET isotherm for liquid phase adsorption [40], where *k*_BET(s)_ applies to the equilibrium constant of adsorption of the first layer and *k*_BET(L)_ refers to the equilibrium constant of adsorption of upper layers.

The values of *q* and *q_t_* were calculated by the equations (1) and (2):


(1)

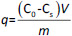




(2)

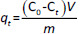



where *C*_0_ is initial concentration of the solution (mg L^-1^), *C_t_* is concentration of the solution in time *t* (mg L^-1^), *V* is volume of solution (L) and *m* is adsorbent mass (g).

Equation (3) was used for the adsoprption (%) calculation:


(3)

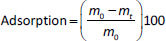



where *m*_0_ - the initial mass of the adsorbate, *m_t_* - is the adsorbate mass after time *t*.

### Determination of the effect of MB adsorption on the pH of starch

The effect of MB adsorption on starch pH was investigated. The suspensions of pure starch in distilled water and suspensions of starch with adsorbed MB were prepared according to adsorption conditions (50 mg starch per 40 mL of water). pH measurements were taken after 1 and 72 h at 22±2 °C. Each measurement was taken three times. An ANOVA test with *α* = 0.05 was performed for these measurements.

## Results

**Figure 2.**
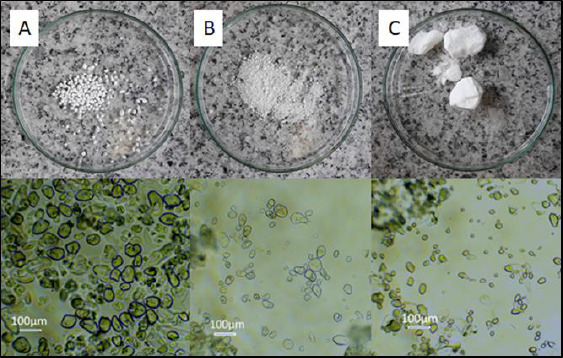
Images of actual appearance (above) and images magnified 10 times with a stereoscopic microscope (SMZ-171-TLED, Motic, Hongkong, China) (below) of A - SM1, B - SM2, C - SM3; petri dish diameter = 10 cm.

The results of the starch synthesis procedures yielded products with structures that conformed to the patterns (Figure 1 in the Introduction), and the appearance was as shown in Figures 2A and 2B.

The molar ratio of functional groups added to the modified starches was converted per 1 g of native starch.

### Spectroscopic evaluations

#### FTIR spectroscopy

The FTIR spectrum of pure MB showed a broad peak from 3219 to 3364 cm^-1^, indicating an O-H bending bond. The sharp peaks at 3050 cm^-1^ and in the range of 817 to 884 cm^-1^ were responsible for the presence of C-H or =C-H bonds in the aromatic ring. Similarly, a sharp peak around 2700 cm^-1^ may have occurred due to the C-H stretching bonds [41].

**Figure 3.**
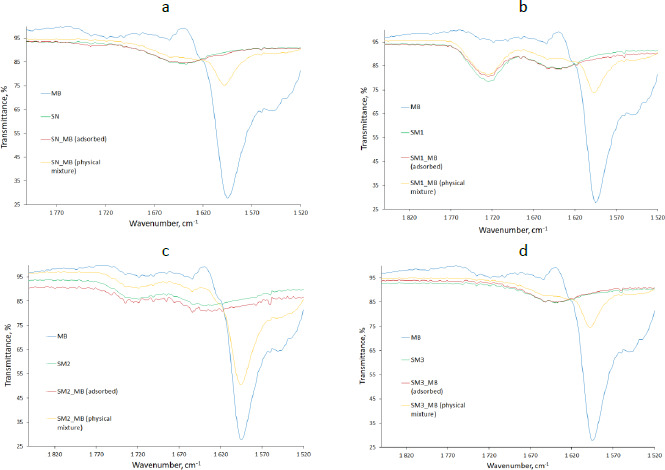
The FTIR spectra of methylene blue (MB), the pure starches (a) SN, (b) SM1, (c) SM2 and (d) SM3, the physical mixture and the experimental formulation of the starches with adsorbed MB, respectively.

Characteristic peaks interacting at 1596 cm^-1^ might have been assigned to N=C stretching bonds. The peaks in the range 1420-1490 cm^-1^ most likely belonged to the stretching C=C-C bonds in the aromatic ring. Vibration from 1340 to 1360 cm^-1^ revealed the presence of C-N stretching bonds in aromatic tertiary amine [41] and peaks from 1059 to 1064 cm^-1^ and 661 cm^-1^ are related to C-S-C stretching bonds in heterocycle [42]. The pure starches FTIR spectra indicated a broad peak of O-H stretching bond at about 3300 cm^-1^, C-O stretching vibration at the range 1185-1347 cm^-1^, especially the strong peak at 1240 cm^-1^ for SM1 (Figure 3 b), C-O stretching bond at 930 cm^-1^ [43] and O-H bending bond at 988 cm^-1^ [44] (Figure 3).

The plot of the spectrum of adsorbed MB on the starch (red line) mostly followed the plot of the spectrum of the respective starch in its pure state (green line). However, there were locations where individual peaks were in excess. The peaks assigned to N=C stretching bonds at about 1600 cm^-1^ appeared in the case of MB adsorbed on the SN and SM2, however, to a very slight extent (Figure 3 a,c). The peak indicating the presence of the C=O bond of the acetyl group at 1730 cm^-1^ [45] was spotted on the SM1 spectra (Figure 3b).

### PXRD

**Figure 4.**
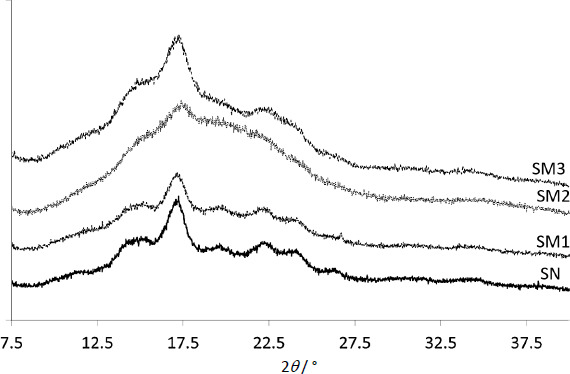
The diffractograms of acetylated starch (SM1), starch citrate (SM2) and starch diphosphate (SM3) compared to native starch (SN).

Diffractograms of modified starches were compared to those of SN (Figure 4). The peaks with the highest intensity were in the 2 range from 17.2 to 17.4°. The most intense belonged to SN and the least to SM2.

The proportion of crystallinity and amorphousness for the tested samples was determined using the amorphous subtraction method [46,47] and presented in Table 3.

### Thermal properties

**Figure 5.**
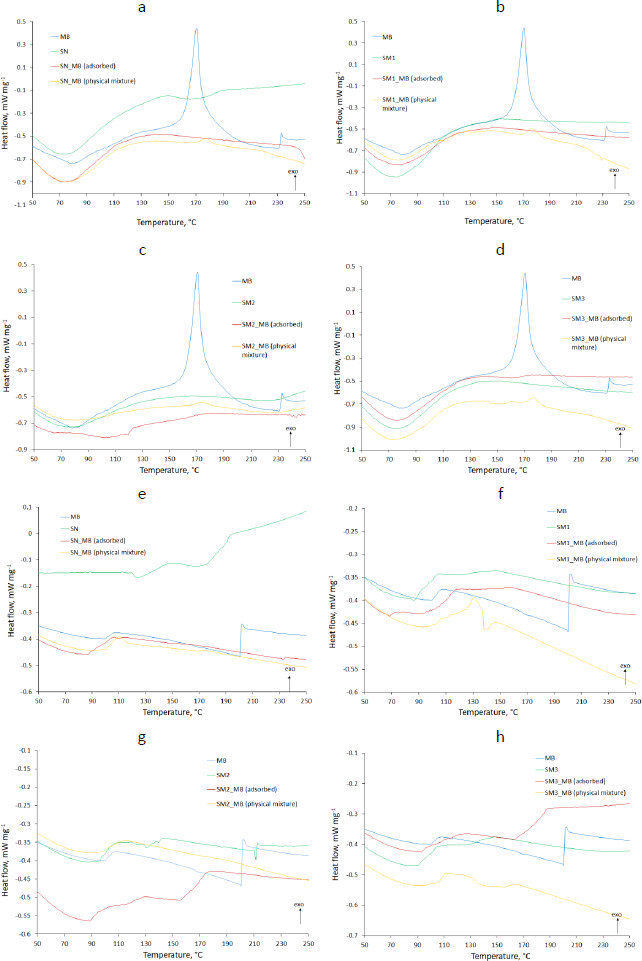
The DSC thermograms of methylene blue (MB), the pure starches (SN, SM1, SM2 and SM3), physical mixtures, and the experimental formulations of the starches with adsorbed MB. The subfigures (a,b,c,d) represent thermograms of the first heating cycle and (e,f,g,h) thermograms of the second heating cycle.

DSC analysis results shown in Figure 5 compared pure MB and evaluated starches with adsorbed MB and physical mixtures of these components. The thermograms showed the results of heat flow measurements from 0 to 350 °C taken in two cycles, with a cooling interval in between. The exothermic direction has been marked with an appropriate arrow (*exo*).

**Figure 6.**
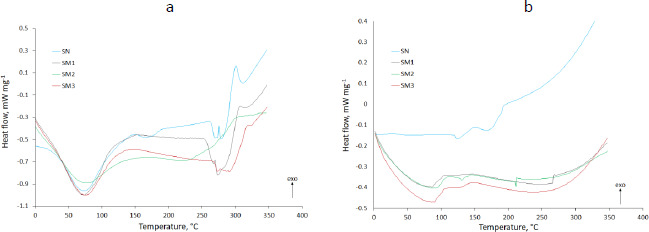
The DSC thermograms of the starches (SN, SM1, SM2 and SM3). The subfigure (a) represents thermograms of the first heating cycle and (b) the second heating cycle.

The results of DSC testing of native and modified starches under two heating cycles are shown in Figure 6.

The glass transition midpoint temperatures *T*_g_, the difference in the heat capacity between the transition from glass to the liquid state Δ*C*_p_ and Δ*T* - difference between onset crystallization temperature and *T*_g_ were analyzed using Proteus 7.0 software (Netzsch, Selb, Germany). The results are shown in the Supplementary material, Table S1.

### Adsorption tests

**Figure 7.**
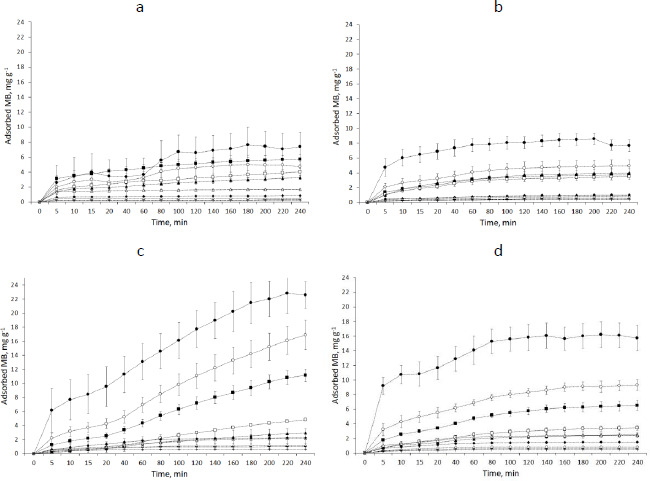
The plot of MB adsorption on the starch samples with time: (a) SN, (b) SM1, (c) SM2, (d) SM3; • - 2 mg, ◦ -5 mg, ▪ - 10 mg, □ - 25 mg, ▲ - 50 mg, Δ - 125 mg, ♦ - 250 mg, ◊ - 500 mg, **-** - 750 mg, + - 1000 mg, *n* = 4, at 22±0.5 °C and 6.0 mg L^-1^ MB solution with pH 4.26.

The graphs showing the course of MB adsorption on starch showed the lowest saturation for SN and the highest for SM2 for 2 mg starch samples. It was 7.60 mg g^-1^ for SN and 22.86 mg g^-1^ for SM2 (Figure 7).

The amount of adsorbed MB increased with the enhancement of the mass of starch samples. The parameter values, adjusted to the tested kinetics models, based on the correlation factor, are given in the Supplementary material, Table S2.

Table 4 exhibits the parameters adapted to the chosen isotherm models. The samples of the starches were divided into two categories dependent on the mass; “A” was between 2 and 125 mg and “B” was between 250 and 1000 mg. Freundlich isotherm described in a satisfactory way adsorption equilibriums of MB adsorbed on the SN A, SM1, SM2 and SM3 B. The Freundlich exponent 1/*n* between 0.5723 and 0.9221 for SN “A” and SM1 “B” indicated favorable adsorption for which 1 ≤ 1/*n*. The adsorption on SM2 “B” had the largest value of k_F_. The adsorption on SM1 “A” had the lowest *k*_F_-value. In the case of the Langmuir isotherm, only SM2 “B” and SM3 “B” gave a satisfactory value of *r*^2^. However, in this case, the values of *q*_m_ and *k*_L_ were negative. The largest *r*^2^ values for the BET isotherm belonged to SM2 “B” and SM3.

### Determination of the effect of MB adsorption on the pH of starch

**Figure 8.**
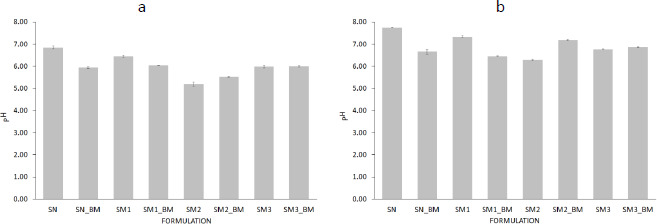
Comparison of pH values of starch suspensions with adsorbed MB to pure starch suspensions after (a) 1 h and (b) 72 h at 22±2 °C (*n* = 3).

The pH of starch suspensions with adsorbed MB compared to that of pure starch suspensions was higher for SM2 and lower for SN and SM1. In addition, there was a proportional increase in pH values after 72 h (Figure 8).

No significant differences were observed between the pH values of SN-MB and SM1-MB after 1 h (Figure 8a).

## Discussion

### Spectroscopic evaluations

#### FTIR spectroscopy

Due to the appearance of peaks at 1599 cm^-1^ assigned to the C=N bond on the FTIR spectrum of samples of adsorbed MB on SN and SM2, we postulate the existence of an interaction between the N^+^ ion derived from MB and the OH^-^ group belonging to starch, which probably leads to enhanced adsorption of the dye on the polymer. This observation coincides with the mechanism of MB adsorption on black olive stone carried out by Al-Ghouti *et al.* [48].

#### PXRD

According to Table 3, the SN was the most crystalline, and SM2 the least. The crystallinity of SM increased according to the following pattern, SM2<SM3<SM1<SN, which suggests that the implementation of ionic groups resulted in the highest amorphicity, whereas the non-ionic acetyl groups in the lowest extent influenced the increase of amorphicity. This remark agrees with the results of Bhargav *et al.* [49], who assessed the combination of PVA with NaI. As the salt content of the polymer increased, the DSC diffraction peaks became less intense. They suggested high susceptibility of the amorphous structure of the polymer to ionic diffusivity of ionic particles. Similarly, Hodge *et al.* [50] found a correlation between peak intensity and the degree of crystallinity. They observed that the addition of water resulted in a decrease in XRD peak intensity and an increase in PVA amorphousness.

### Thermal evaluation

#### DSC

The DSC thermograms of pure MB in the first heating cycle displayed an endothermic peak at about 80 °C responsible for the dehydration (Figure 5 a-d). The thermogram of the second heating cycle no longer showed this peak, while an endothermic peak in the 100 °C range appeared (Figure 5 e-h). MB forms several hydrates. In addition, it reacts with an aluminum container for DSC [51]. This endothermic peak may indicate a transition from one form of MB hydrate to another. In the thermogram of the first heating cycle, it was most likely obscured by the broad peak of dehydration. These speculations are confirmed by the shifted exothermic peaks occurring at 171 and 233 °C in the first heating cycle (Figure 5 a-d) and 201 °C in the second cycle (Figure 5 e-h). They testify to the decomposition of MB [52]. Omer *et al.* [53] indicated the occurrence of oxidation of MB at 260 °C. A difference was observed between the spectrum of adsorbed MB and physical mixed MB. An exothermic peak appeared, indicating the presence of MB in the physical mixture with SN (177.6 °C), SM1 (175.9 °C), SM2 (174.0 °C) and SM3 (177.2 °C) (Figure 5 a,b,c,d respectively). No MB peak was observed in MB adsorbed at SN and SM1 (Figure 5 a,b), which may indicate the absence or complete decomposition of MB. For MB adsorbed on SM2 and SM3, the course of these peaks was flattened at 174.4 and 176.7 °C, respectively, which may indicate the interaction of MB with these starches (Figure 5 c,d).

Glass transition temperature *T*_g_ value readings showed the lowest *T*_g_ for SM2 (47.2 °C) and the highest for SM3 (55.9 °C) (Table S1). Hydrogen bonds are among the factors affecting *T*_g_ [54]. Taylor *et al.* [55] observed significantly lower *T*_g_ of sucrose compared to other disaccharides. This was due to the less extensive hydrogen bonding network in the amorphous state. Similarly, Zhou *et al.* [56] demonstrated the dependence of *T*_g_ on hydrogen bonds occurring between polymer and copolymer particles (maleimide-isobutene alternating copolymers). A network of hydrogen bonds of carboxyl groups resulted in a higher *T*_g_ value than hydrogen bonds between hydroxyl groups. The lowest *T*_g_ occurred in the absence of hydrogen bonds between polymer molecules. In our case, the holders of the hydroxyl groups were SN and SM1 (*T*_g_ 52.2 and 51.15 °C respectively) (Table S1) and the carboxyl groups were present in the SM2 molecule. In addition, phosphate groups appeared in the SM3 molecule. High *T*_g_ values for SM3 may suggest the self-association of these starch molecules, whose *T*_g_ decreased to 53.0 °C after bond formation with MB [55]. In the case of SN, *T*_g_ values increased after MB adsorption (54.7 °C), what may indicate low self-association of SN particles in the pure state.

In the present work, the values of Δ*T* were assessed to determine the thermal stability of the tested samples. The higher the value of Δ*T*, the better the thermal stability. The value for SN was the highest (221.07 °C), demonstrating the highest thermal stability of this starch. At the same time, its highest percentage of crystallinity was confirmed (Table 3). On the contrary, SM2 showed the lowest value of Δ*T* (179.8 °C) - the lowest thermal stability. Keshk *et al.* [43] demonstrated the dependence of thermal stability on Δ*T*-values, which correlated with the degree of crystallinity of corn starch-cellulose mixtures, depending on the mixture ratio. They found an inversely proportional relationship between Δ*T* values and Δ*C*_p_ values. Our Δ*C*_p_ studies confirmed such a relationship outside of the SM2 samples having the lowest Δ*C*_p_ value (0.465 J g^-1^·K^-1^) according to the lowest Δ*T* (Table S1). This phenomenon may be supported by the observation of Liu *et al.* [57]. They studied various starches whose structure was more or less amorphous. According to them, amorphousness gives the compounds a liquid-like structure. More amorphous compounds have a less ordered structure than those with a crystalline structure. They showed that the process of lowering the temperature below *T*_g_ leads to the reorganization of the structure. In our study, SM1, SM2 and SM3 starches were hardened at 2 degrees below *T*_g_. Moreover, the research is confirmed by PXRD studies, where SM2 had the highest degree of amorphousness (Table 3).

The thermograms of the second heating cycle can show the structure of the compound when structural reorganization occurs after the cooling cycle. In the case of SM2 there was an endothermic peak at 215 °C, from which an increase in heat flow is observed. The thermograms of adsorbed MB on starches are characterized by different temperatures of the start of the exothermic process. The increase in heat flow started fastest for SM2_MB (adsorbed) at 157 °C and slowest for SM3_MB (adsorbed) at 163 °C (Figures 5g and 5h, respectively). This endothermic event might be attributed to reduced hydrogen bonding as well as the interference of molecular organization due to the interaction between SM2, SM3 and MB. For SN_MB (adsorbed) and SM1_MB (adsorbed), no increase in heat flow was observed (Figures 5e and 5f).

#### Adsorption tests

MB adsorption on starch predominantly matched a pseudo-second-order model. A study by Lin *et al.* [58] of MB adsorption on activated carbon indicated a better pseudo-second-order fit to both linear and nonlinear models. Similarly, Ma *et al.* [45] matched the adsorption of heavy metal ions on modified potato starch to the pseudo-second-order model. In our study, the highest *k*_2_ values obtained for SN, SM3, SM2 and SM1 were 1.9623, 1.5367, 0.9240, 0.1848 g mg^-1·^min^-1^, respectively, and occurred at a weight of 1000 mg for SN and SM1 and 2.0 mg for SM2 and SM3 (Table S2). This suggests that MB adsorbed fastest on SN, but this was dependent on the surface area of the adsorbent: the larger the surface area, the faster the saturation. In the case of SM2 and SM3, MB adsorbed fastest already on the smallest starch particle, suggesting a high affinity of the adsorbate to the adsorbent. The slowest adsorption was observed in SM1.

**Figure 9.**
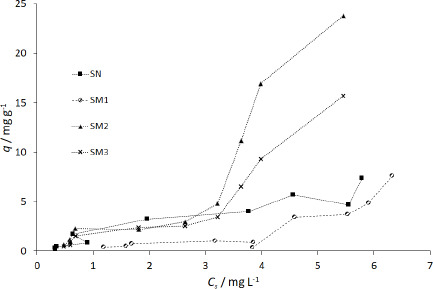
Isotherms of MB adsorbed on the starches: SN, SM1, SM2 and SM3 at 22±0.5 °C and 6.0 mg L^-1^ MB solution with pH 4.26 (*n* = 4).

The *q* values in Table S2 differ from *q*_m_ obtained according to Langmuir and BET isotherms. Moreover, some of the Langmuir adsorption constants (*q*_m_ and *K*_L_) showed negative values (Table 4). Langmuir isotherm is known to get different parameters according to the calculation method [59-61], therefore, we used the course of the isotherm of MB on the starches to establish *q*_m_ values (Figure 9).

Methylene blue is a substance that has been thoroughly studied by scientists because of its harmfulness to the environment. As a dye used in industry, it leaks into water waste in large quantities. Their purification is a major challenge for researchers. Many study substances that are adsorbents for MB and can serve as filters [32,61-65]. Some of these include starches and other polysaccharides. Table 5 shows the *q*_m_ values of example adsorbents for MB.

The sigmoidal course of the SM2 and SM3 starch isotherms falls into type IV according to the IUPAC classification. SN and SM1 isotherms are more aligned with type VI [76]. Type IV represents single and multilayer adsorption plus capillary condensation. Type VI illustrates that the adsorption isotherm can have one or more steps. In our study, the adsorptions on SN and SM1 starches best fitted to the Freundlich isotherm model. On the other hand, for SM2 and SM3 starches, the adsorption courses more closely matched the BET isotherm (Table 4).

The Freundlich model describes multilayer adsorption on a surface, with an inhomogeneous distribution of binding sites and, therefore, an inhomogeneous energy distribution. The constant *k*_F_ expresses the maximum adsorption on the surface of the adsorbent. The BET isotherm describes the phenomenon of physisorption on a non-microporous surface. The constant *k*_BET_ is related to the adsorption energy. Bhattacharyya *et al.* [36] found graphene oxide-potato starch almost perfectly fitted to the Freundlich isotherm model. Dipa *et al.* [37] adapted MB adsorption on kaolinite to the Langmuir model. Bestani *et al.* [62] studied MB adsorption on carbon-activated leaves, which they fitted to the Langmuir and Freundlich models. El Qada *et al.* [31] matched MB adsorption on activated carbon to Redlich-Peterson and Langmuir isotherm models. The Redlich-Peterson model is a compilation of the elements in the equations of the Langmuir and the Freundlich isotherms. Langmuir isotherm mostly applies to chemisorption on a monolayer. Activated carbon is highly porous and is a well-known adsorbent used in industry as a filter [77]. The dependence of the isotherm model on the structure of the adsorbent can also be supported by the study of Ma *et al.* [45], who used potato starch modified with high temperature to increase its porosity. Adsorption of heavy metal ions on such modified starch showed good adherence to the Langmuir model. In the case of our experiment, only SM2 was subjected to high-temperature modification at 100 °C. This could most likely increase its adsorption properties.

### Determination of the effect of MB adsorption on the pH of starch

The measured pH and p*K*a values of the acids from the literature enabled us to estimate the number of moles of functional groups derived from the molecules and finally present at the modified starches. The calculated values are gathered in Table 6.

The initial assumptions of the ratio of the number of millimoles of functional groups on the modified starches used to react with the native starch during its modification was 23:13:1 for SM1:SM2:SM3, respectively (Table 1). The millimole ratio of the same groups, calculated from the pH values of the modified starch suspensions after 1 h, was 1:13:0.03. This shows that the activity of acyl and phosphate groups decreased 23 and 33 times compared to carboxyl groups, respectively. MB solution with pH of 4.26 resulted in the acidification of SN and SM1 suspensions. It did not change the pH for SM3. The increase in the pH value of the suspension of adsorbed MB on SM2 starch may indicate the most effective binding of MB to the carboxyl groups of SM2 (Figure 9).

### Effect of type of functional groups of modified starches on interactions with MB

The MB adsorbed on the 1000 mg of the starch decreased according to the following pattern: SN > SM2 > SM3 > SM1 (Figure 7). The hydroxyl, carboxyl and phosphate groups of starch favored MB binding to the polymer to the greatest extent, while the acetyl groups had the greatest effect on the reduction of adsorption capacity. The rationale for the decreased sorption capacity could be the blocking of starch functional groups in the structure of starch polymer or the specific activity of functional groups towards MB. Dipa *et al.* [37] observed the effect of NaOH-derived hydroxyl groups on the increase of MB adsorption on kaolinite. However, these were groups added to kaolinite. In our case, only the carboxyl, phosphate and acyl groups originated from acids and were added during the starch modification process. Physisorption may be taken into account in the case of SN and SM3. It differs for SM2 and SM1, where acid groups can affect MB adsorption. Similar conclusions were issued by Huang *et al.* [80] after studying the adsorption of Pb^2+^ ions on activated carbon with additional functional groups. Pb^2+^ ions were adsorbed according to three mechanisms: (1) in mesopores, (2) *via* bonds between -OH and C=O and Pb^2+^, (3) H^+^ from the acidic carboxyl and phosphate groups is replaced by the Pb^2+^ ion.

An additional element worth highlighting is the effect of the method of preparation of modified starches on their structure. Two of our SM2 and SM3 starches were prepared using the crosslinking method, which provides increased starch stabilization by forming bridges between hydroxyl groups and anhydroglucoses [81]. Kapelko *et al.* [17] found crosslinking reactions caused a decrease in solubility in water and a decrease in phase transition. Shen *et al.* [82] used corn starch and citric acid, malic acid, succinic acid and 1,2,3,4-butanetetracarboxylic acid (BTCA) as cross-linkers. They observed that the acid with more carboxyl groups (citric acid and BTCA) showed higher crosslinking degrees and enhanced mechanical properties than acids with two carboxylic groups. Moreover, as they are used in food modification, poly-carboxylic acids are in the category of safe chemicals [83]. In the case of SM1 and SM3, the chemically modified starches with a low degree of substitution (DS) are permitted for use in the food industry and are denoted with the symbol “E” followed by an appropriate number. Among others, acetylated starch (E1420) and di-starch phosphate (E1412) belong to this group [84].

## Conclusions

Citrate starch (SM2) presented the best adsorption capacity, followed by phosphate starch (SM3), and acetylated starch (SM1) showed the weakest adsorption capacity, according to the adsorption results. MB adsorption on SM2 and SM3 followed the BET isotherm model, which shows the physical adsorption of the cationic dye on the low-porous surface. In the case of SM1, MB adsorption probably took place *via* electrostatic attraction between the heterogeneous adsorbent surface and the adsorbate molecules, as indicated by the Freundlich adsorption model.

Spectral methods confirmed the adsorption capacity of SM2. In the FTIR study, a probable interaction between the OH^-^ groups of SM2 starch and N^+^ of MB was revealed on the basis of characteristic vibration of the C=N bond in the adsorbent derived from MB at 1600 cm^-1^.

The DSC results showed the lowest thermal stability of SM2 correlated with its amorphous structure. Moreover, according to DSC, the possibility of interaction between SM2 or SM3 and MB was demonstrated *via* the existence of an exothermic peak belonging to MB in samples of these two adsorbents. The appearance of endothermic peaks in the case of SM2 and SM3 samples with adsorbed MB in the second heating cycle confirmed the interaction between the adsorbents and the adsorbates. The effect of the manufacturing method of the modified starches on their durability was noted. SM2 and SM3 starches were prepared using the crosslinking method, which provided increased starch stabilization.

The above presented properties and, in addition, the “safe chemistry” and slightly acidic pH of the tested starches make them useful, especially citrate starch, as carriers for biologically active substances containing cationic groups, in topical skin applications. The present research confirms our previous studies on lidocaine hydrochloride. The modified starches may be the potential carriers in the form of hydrogels, similar to the hydrocolloid dressings.



## Figures and Tables

**Table 1.** Number of moles of individual functional groups of modified starches per 1 g of SN.

Modified starch	Concentration, mmol g^-1^		
	Carbonyl groups	Carboxyl groups	Phosphate groups
SM1	2.74	-	-
SM2	-	1.56	-
SM3	-	-	0.12

**Table 2.** Isotherm and kinetics models applied for evaluation of obtained data, *C*_s_ - concentration of substance in the solution in the equilibrium state (e.s.), *q*_*t*_ - adsorbed quantity of adsorbate in time, *q* -adsorbed quantity of adsorbate in the e.s., *q*_m_ - maximum monolayer capacity, *k*_*F*_, *k*_*L*_ and *k_BET_* - adsorption equilibrium constants, *k*_*1*_, *k_2_* - equilibrium rate constants, 1/*n* - constant, *t* - time, *a* - slope, *b* - intercept, *r*^2^ - regression coefficient.

Applied model	General equation	Parameters			
Freundlich	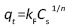	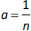	*b*=In*k*_F_	-	*r^2^*
Langmuir	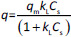	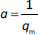	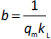	*-*	*r^2^*
BET	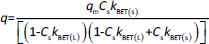	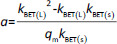	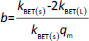	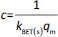	*r^2^*
Pseudo - 1^st^ order	ln(*q*-*q_t_*) = ln*q* - *k*_1_*t*	*q*	*k_1_*	*-*	*r^2^*
Pseudo - 2^nd^ order	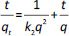	*q*	*k_2_*	*-*	*r^2^*

**Table 3.** Composition of crystallinity and amorphousness for SM1, SM2 and SM3 samples compared to SN (*n* = 3).

Starch types	SN	SM1	SM2	SM3
Crystallinity, %	14.3 ± 0.80	9.8 ± 0.14	3.2 ± 0.26	5.7 ± 0.75
Amorphousness, %	85.7 ± 0.80	90.2 ± 0.14	96.8 ± 0.26	94.3 ± 0.75

**Table 4.** Parameters of selected isotherm models for adsorption of 6 mg L^-1^ MB solution on SN, SM1, SM2 and SM3 starches. A - applies to 2 to 125 mg, B - applies to 250 to 1000 mg of the adsorbents (*n* = 4).

Isotherm model	Parameters	SN		SM1		SM2		SM3	
		A	B	A	B	A	B	A	B
Freundlich	1/*n*	0.5723	1.0254	4.8442	0.9221	2.4503	3.5491	2.4769	1.5960
	*k*_F_ / L g^-1^	2.1632	0.9702	0.0010	0.3727	0.3964	7.7983	0.1407	0.5681
	*r* ^2^	0.9071	0.8096	0.8467	0.8834	0.8954	0.9162	0.9083	0.9981
Langmuir	*k*_L_ / L g^-1^	0.3020	-0.0712	-0.1593	0.0488	-0.1671	-1.2145	-0.1317	-0.2738
	*q*_m_ / mg g^-1^	8.9767	-12.594	-0.3920	7.9177	-4.7619	-0.4387	-3.4807	-1.5094
	*r* ^2^	0.6793	0.0771	0.5560	0.0836	0.7378	0.9220	0.8701	0.9684
BET	*k*_BET(s)_ / L mg^-1^	1.4106	4.7971	0.5151	0.7223	0.0216	5.1912	1.8188	0.7352
	*k*_BET(L)_ / L mg^-1^	0.0821	0.6768	0.0741	0.1666	0.1399	1.2943	0.1539	0.2591
	*q*_m_ / mg g^-1^	3.3822	0.0318	0.0290	0.3175	27.676	0.2730	1.4069	0.4535
	*r* ^2^	0.7331	0.3582	0.6907	0.2395	0.7706	0.9334	0.9434	0.9813

**Table 5.** Maximum adsorption capacities (*q*_m_) of different starches and other polysaccharides toward MB.

Type of adsorbent	*q*_m_ / mg g^-1^	Ref.
Hydrogel poly(sodium methacrylate) with wheat starch and eggshell	1.95	[66]
Crosslinked porous corn starch	9.46	[67]
Starch cryogel of a rice flour and a tapioca starch	34.84	[68]
Honeycomb biomass adsorbent based on oxidized corn starch-gelatin	1551.5	[69]
Hydrogel poly(acrylamide) with cassava starch	1917	[70]
Hydrogel poly(acrylic acid) with potato starch crosslinked with N,N’-methylene-bisacrylamide	2967.66	[71]
Xanthate modified magnetic chitosan	197.8	[72]
Chitosan/activated charcoal	500	[73]
Cellulose-grafted poly 4-hydroxybenzoic acid magnetic nanohybrid	7.5	[74]
Hydrogel of hydroxypropyl cellulose (HPC) composited with graphene oxide	6.59	[75]
Native potato starch	7.60	This work
Acetylated potato starch	8.57	This work
Diphosphate potato starch	16.10	This work
Citrate potato starch	22.86	This work

**Table 6.** Concentrations of functional groups located on 50 mg of modified starches recalculated on the basis of parameter values of the Henderson-Hasselbalch equation [78] (*n* = 3).

	SM1	SM2	SM3
pH after 1h	6.44 ± 0.05	5.19 ± 0.09	5.98 ± 0.07
p*K*_a1_*	4.76	3.13	2.16
p*K*_a2_*	-	4.76	7.21
p*K*_a3_*	-	6.40	12.32
Concentration of functional groups, mmol mg^-1^	6.07 ± 1.64 ×10^-7^	7.07 ± 2.82 × 10^-6^	1.90 ± 0.62 × 10^-8^

*according to database in LibreTexts™ chemistry [79]
