# Supplementary material for: The interactions of model cationic drug with newly synthesized starch derivatives
Source: ADMET DMPK. 2023 Sep 20;11(3):387–407. doi: 10.5599/admet.1950 (PMC10567071; doi:10.5599/admet.1950)
Supplement: Supplementary file 1 [file ADMET-11-1950-S1.pdf]

ADMET & DMPK 11(3) (2023) S11-S12

Open Access : ISSN : 1848-7718

http://www.pub.iapchem.org/ojs/index.php/admet/index

Supplementary material to

The interactions of model cationic drug with newly synthesized starch derivatives

Justyna Kobryń[1], Tomasz Zięba[2], Magdalena Rzepczyńska[1] and Witold Musiał[1]*

[1]*Department of Physical Chemistry and Biophysics, Wrocław Medical University, Borowska 211A, 50-556 Wrocław, Poland*

[2]*Department of Food Storage and Technology, Faculty of Biotechnology and Food Science, Wroclaw University of Environmental and Life Sciences, Chełmońskiego 37, 51-630 Wrocław, Poland*

ADMET & DMPK 11(3) (2023) 387-407; https://doi.org/10.5599/admet.1950

Thermal properties

Table S1. The DSC parameters of methylene blue (MB), the pure starches (SN, SM1, SM2 and SM3), physical mixtures and the experimental formulations of the starches with adsorbed MB.

	T_g / °C	ΔC_p / J g^{-1}·K^{-1}	ΔT / °C
MB	40.8	0.621	190.20
SN	52.20 ± 2.55*	1.598 ± 0.331*	221.07
SN_MB (adsorbed)	54.7	0.721	202.75
SM1	51.15 ± 6.29*	1.862 ± 0.733*	216.36
SM2	47.2	0.465	179.80
SM3	55.9 ± 1.77*	1.717 ± 0.261*	217.76
SM3_MB (adsorbed)	53.0	1.348	218.67

*value of two DSC measurements

Adsorption tests

The majority of starch formulations showed the best fit to the pseudo-second order kinetic model (Table S2). Based on the values of MB mass (mg) adsorbed on 1 g of starch, the *q* values presented the adsorption capacity. These values decreased in the following order: SM2 > SM3 > SN > SM1. No significant difference was observed between 2 and 5 mg of SM1, 250 mg of SM2 and 10 mg of SM3 and the reference SN in the respective weights.

Table S2. The parameters established in kinetics models of adsorption of MB on the SN, SM1, SM2 and SM3: *q* - measured quantity of adsorbed substance in e.s., $k_{1,2}$ - rate constant for pseudo first and second order, respectively, r^2 - determination coefficient (n = 4).

Adsorption kinetics model	Starch weight, mg	Pseudo first-order parameters			Pseudo second-order parameters		
		q / mg g^{-1}	k_1 / min^{-1}	r^2	q / mg g^{-1}	k_1 / min^{-1}	r^2
SN	2	4.3618	0.0125	0.7950	11.473	0.0006	0.3388
	5	3.3970	0.0230	0.9490	5.0936	0.0092	0.9838
	10	2.8844	0.0153	0.9589	5.7903	0.0150	0.9970
	25	2.5508	0.0113	0.9321	4.0287	0.0121	0.9841
	50	2.0487	0.0121	0.9361	3.3082	0.0155	0.9887
	125	0.3742	0.0153	0.8697	1.6975	0.1628	0.9996
	250	0.3323	0.0141	0.8994	1.0059	0.1691	0.9987
	500	0.0649	0.0195	0.8447	0.4572	1.3930	0.9999
	750	0.0482	0.0188	0.8631	0.2990	1.7957	0.9999
	1000	0.0398	0.0175	0.8460	0.2298	1.9623	0.9999
SM1	2	1.9249	0.0091	0.4279	8.1079	0.0489	0.9931
	5	3.0101	0.0211	0.9890	4.9831	0.0162	0.9992
	10	2.4687	0.0192	0.9794	3.8840	0.0181	0.9993
	25	2.3265	0.0185	0.9675	3.6039	0.0179	0.9994
	50	1.4945	0.0174	0.9672	1.4559	0.0408	0.9989
	125	0.9384	0.0201	0.9728	0.9262	0.0853	0.9991
	250	0.7467	0.0174	0.9845	1.1002	0.0489	0.9956
	500	0.5549	0.0180	0.9791	0.7843	0.0668	0.9978
	750	0.3570	0.0162	0.9411	0.5278	0.1083	0.9951
	1000	0.2363	0.0156	0.9690	0.4282	0.1848	0.9982
SM2	2	21.018	0.0118	0.9642	29.386	0.9240	0.9677
	5	17.839	0.0114	0.9267	21.334	0.4760	0.9071
	10	12.734	0.0125	0.8890	14.715	0.3832	0.8945
	25	5.6116	0.0114	0.9421	12.925	0.1793	0.7890
	50	3.1886	0.0122	0.9386	3.8714	0.2019	0.9531
	125	3.4664	0.0187	0.9903	4.2264	0.3624	0.9981
	250	1.9393	0.0236	0.9625	2.3046	0.2681	0.9899
	500	0.6607	0.0272	0.9858	1.0215	0.2885	0.9999
	750	0.3280	0.0337	0.9825	0.7218	0.3604	0.9998
	1000	0.2751	0.0313	0.9777	0.5589	0.2911	0.9999
SM3	2	6.9662	0.0193	0.8920	16.373	1.5367	0.9987
	5	6.8448	0.0185	0.9726	9.8658	0.7272	0.9969
	10	5.1754	0.0177	0.9869	6.9897	0.5518	0.9970
	25	2.8545	0.0173	0.9845	3.7589	0.3788	0.9978
	50	1.7755	0.0163	0.9871	2.6564	0.3689	0.9973
	125	1.2140	0.0231	0.9815	2.5056	0.4924	0.9999
	250	0.8265	0.0238	0.9533	1.5485	0.4336	0.9999
	500	0.3877	0.0272	0.9565	0.8979	0.3703	0.9999
	750	0.2391	0.0266	0.9495	0.6295	0.3350	0.9999
	1000	0.2003	0.0264	0.8875	0.5116	0.2722	0.9997

CC BY
